# A Case of Atrial Flutter Masking Acute Pericarditis

**DOI:** 10.7759/cureus.14168

**Published:** 2021-03-29

**Authors:** Christopher Schwartz, Arjun C Khadilkar, Christopher Bitetzakis, Aarti Patel

**Affiliations:** 1 Internal Medicine, University of South Florida Morsani College of Medicine, Tampa, USA; 2 Cardiology, University of South Florida Morsani College of Medicine, Tampa, USA

**Keywords:** atrial flutter, acute pericarditis, cardioversion, ekg, supraventricular tachycardia

## Abstract

Atrial flutter (AFL) is a macro-reentrant tachycardia that can be provoked by numerous factors, including acute pericarditis. We present a case of new-onset AFL masking acute pericarditis in a man with multiple comorbid conditions, including hypertension, chronic kidney disease, and obstructive sleep apnea. After a failed attempt of rate control, the patient underwent successful cardioversion, which revealed electrocardiographic findings consistent with acute pericarditis. Colchicine was avoided in the setting of chronic kidney disease and the patient was treated with a steroid taper. Pericarditis is a rare cause of AFL, and this case demonstrates the diagnostic and management considerations for AFL and acute pericarditis.

## Introduction

Atrial flutter (AFL) is an arrhythmia characterized by rapid, regular, atrial depolarizations at a characteristic rate of between 250 and 350 beats per minute with a 2:1 relation between atrial and ventricular activity [1]. Typical flutter is defined by a macro-reentrant circuit around the cavotricuspid isthmus (CTI), generally in a counterclockwise rotation. Atypical flutter occurs outside the CTI around areas of atrial scar tissue. On electrocardiogram (ECG), typical p-waves are absent, and the atrial activity is seen in a sawtooth pattern known as “F waves” in leads II, III, and aVF [2]. Clinical presentation is often dependent on the ventricular rate and can lead to dyspnea, fatigue, palpations, and lightheadedness. AFL is uncommon in structurally normal hearts and can be seen in clinical contexts similar to those presenting with atrial fibrillation.

Pericarditis is a known trigger for cardiac arrhythmias [3,4]. It is characterized by pericardial sac inflammation due to numerous causes such as infectious, neoplastic, inflammatory, autoimmune, metabolic, pharmacologic, or toxic. On ECG, there may be widespread concave ST elevations and PR depressions with reciprocity in aVR. Presentation varies depending on the underlying etiology but often includes chest pain characterized as sharp, pleuritic, and improved by leaning forward. A pericardial friction rub may be heard during auscultation [5].

We present a case of an 83-year-old-man with supraventricular tachycardia determined to be AFL status post an adenosine trial. After a failed attempt at rate control, the patient underwent cardioversion. ECG findings post-cardioversion in normal sinus rhythm were consistent with pericarditis, which was likely the cause for his new-onset AFL.

## Case presentation

An 83 year-old-male with hypertension, stage three chronic kidney disease, hypothyroidism, transient ischemic attack, and sleep apnea presented with three weeks of pleuritic chest pain, fatigue, subjective fevers, and progressive shortness of breath. The initial ECG was significant for supraventricular tachycardia interpreted as new-onset atrial fibrillation with a rapid ventricular response.

Labs were negative for electrolyte abnormalities and creatinine was 1.5 mg/dL (baseline 1.5-1.9 mg/dL). Troponin-I was within normal limits at 0.01 ng/mL. Influenza A, B, and respiratory syncytial virus (RSV) nasal polymerase chain reaction (PCR) were negative. Coronavirus 2019 (COVID-19) nasal rapid PCR was negative. Thyroid-stimulating hormone was within normal limits at 1.902 lU/ml. C-reactive protein, fibrinogen, and brain natriuretic peptide (BNP) were all elevated at 8.3 mg/dL, 583 mg/dl, 182 pg/mL, respectively. A trans-thoracic echocardiogram showed a preserved ejection fraction of 55-60% with moderate concentric hypertrophy without pericardial effusion. The patient initially received boluses of intravenous diltiazem and transitioned briefly to a continuous diltiazem infusion, which was discontinued due to hypotension. He received an amiodarone load intravenously for rhythm control and renally dosed apixaban for a CHA₂DS₂-VASc score of five. Short-acting intravenous diltiazem was restarted and up-titrated for adequate rate control with hemodynamic stability.

**Figure 1:**
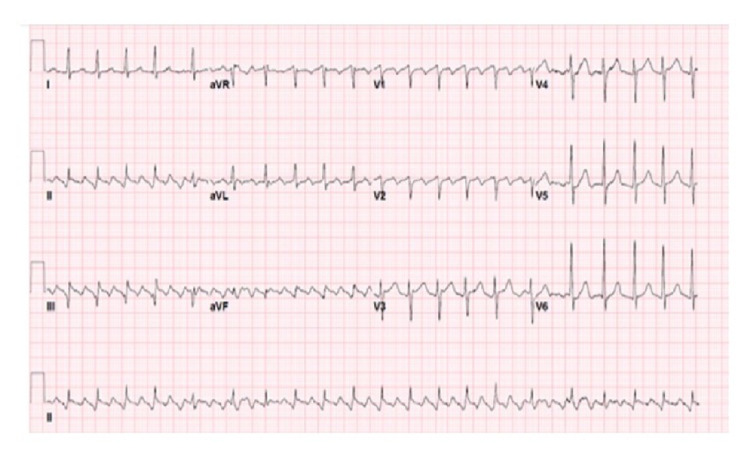
Typical atrial flutter with variable AV block AV block: atrioventricular block [6].

**Figure 2:**
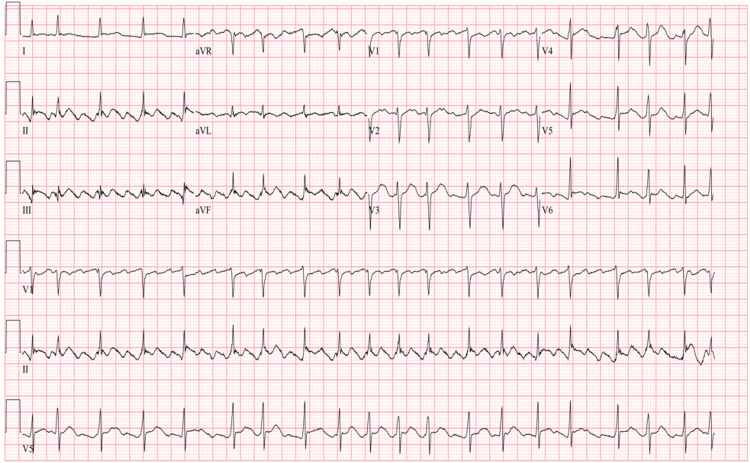
Initial ECG with atrial flutter and variable AV block EKG: electrocardiogram, AV: atrioventricular.

Adenosine was subsequently administered and revealed AFL with variable AV block. A typical atrial activity has been highlighted in Figure 1, and the ECG in Figure 2 highlights the atrial activity in the patient.

**Figure 3:**
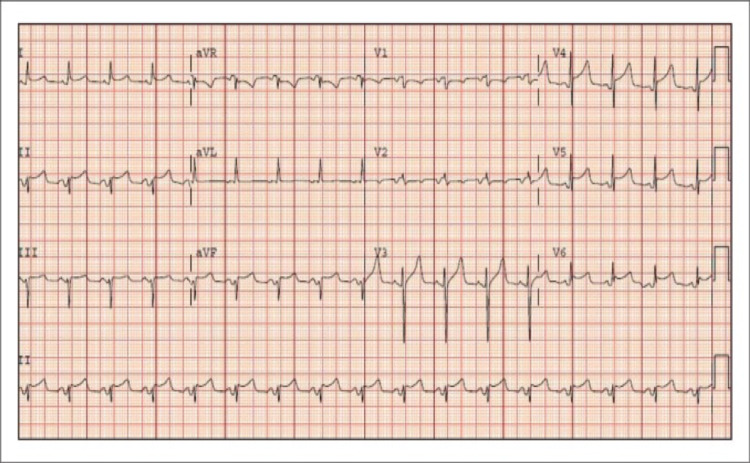
Widespread, concave, ST elevations and PR depressions with reciprocity in aVR aVR: augmented vector right [7].

**Figure 4:**
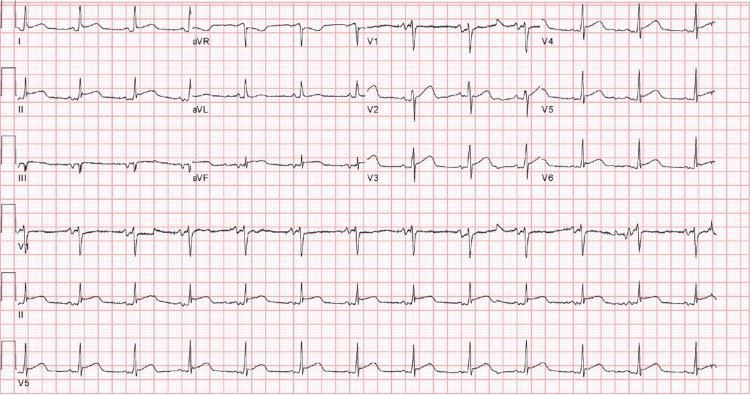
Post-cardioversion ECG consistent with acute pericarditis ECG: electrocardiogram.

Since the patient had persistently increased rates despite initial rate and rhythm control strategies, cardioversion was considered for rhythm control. Intracardiac thrombi were excluded via transesophageal echocardiography and the patient underwent successful electrical cardioversion. Typically, acute pericarditis presents with widespread concave ST elevations and PR depressions with reciprocity in aVR, highlighted in Figure 3. A post-cardioversion 12 lead ECG demonstrated normal sinus rhythm with ST elevations present in the precordial leads with concomitant T-wave abnormalities in V2 through V6, limb leads I, II, and aVL. PR-segment depression was seen in leads I, II, V2, V3, and ST depression in lead aVR (Figure 4).

The patient was asymptomatic post-cardioversion without chest pain, shortness of breath, or diaphoresis. He was diagnosed with pericarditis and treated with a steroid taper due to his chronic kidney disease and history of gastrointestinal bleed in the setting of non-steroidal anti-inflammatory agent (NSAID) use.

## Discussion

In one population-based investigation of AFL, among 58,820 patients in central Wisconsin, those at highest risk for developing AFL were patients with heart failure and chronic obstructive pulmonary disease, with the arrhythmia being uncommon in structurally normal hearts [8]. AFL occurs in similar clinical causes as atrial fibrillation, including but not limited to pericarditis. Though data related to the incidence of arrhythmias in the setting of pericarditis are sparse, atrial fibrillation appears to be the most common across studies occurring at rates ranging from 4.3% to 25% [9]. This wide range is likely due to the various pericarditis etiologies studied and researcher variation in the definition of what constituted a supraventricular arrhythmia. It is unclear why pericarditis predisposes those to arrhythmias though some suggest that the proximity of an inflammatory process to the sinus node may be arrhythmogenic; however, postmortem investigations have challenged this presumption [10].

In the western world, most pericarditis cases are diagnosed as idiopathic after negative work-up, though many of these cases are presumed to be viral [11]. Among immunocompetent patients in developed countries, acute pericarditis is assumed to be of viral etiology with NSAIDs as the mainstay of therapy. Interestingly, the efficacy of NSAIDs has only been evaluated in one randomized control trial with patients suffering from post-pericardiotomy syndrome [12]. Additionally, evidence supports the use of colchicine as an adjunct therapy to NSAIDs to improve remissions rates in both acute and recurrent pericarditis [13].

In those with chronic kidney disease that have contraindications to NSAIDs or colchicine, steroid therapy can be used for the treatment of pericarditis but is associated with disease recurrence. This is especially true early in the disease course as seen in the Colchicine in Addition to Conventional Therapy for Acute Pericarditis (COPE) trial and in a subsequent systematic review where glucocorticoids were a significant predictor of recurrence [13,14]. There is also evidence from animal studies that glucocorticoids may exacerbate virally induced pericarditis by effects on virus replication [15]. Thus, glucocorticoids may perpetuate pericardial inflammation and are considered second-line therapy per the European Society of Cardiology (ESC) [16]. When used, the ESC recommends low to moderate doses such as prednisone 0.2-0.5 mg/kg/day until resolution of symptoms or normalization of C-reactive protein, at which point tapering should begin.

## Conclusions

Atrial fibrillation and AFL are provoked by similar and diverse pathologies including pericarditis. AFL can be managed through pharmacological, electrical, or ablative means. In our case, initial pharmacotherapy treatment failed. After successful electrical cardioversion, a 12-lead ECG demonstrated acute pericarditis, which was the most likely trigger for AFL. Considering his comorbidities of chronic kidney disease and history of NSAID-induced GI bleeding, we concluded that a steroid taper with serial inflammatory laboratory monitoring was the most appropriate therapeutic option moving forward. Pericarditis rarely triggers AFL and, as seen in this case, the classic ECG features of acute pericarditis can be masked during episodes of typical AFL. This case demonstrates the inpatient management considerations for AFL and alternative therapeutic modalities for those with pericarditis and contraindications to first-line therapy.
